# Changes in Cardiac Substrate Transporters and Metabolic Proteins Mirror the Metabolic Shift in Patients with Aortic Stenosis

**DOI:** 10.1371/journal.pone.0026326

**Published:** 2011-10-18

**Authors:** Lisa C. Heather, Neil J. Howell, Yaso Emmanuel, Mark A. Cole, Michael P. Frenneaux, Domenico Pagano, Kieran Clarke

**Affiliations:** 1 Cardiac Metabolism Research Group, Department of Physiology, Anatomy and Genetics, University of Oxford, Oxford, United Kingdom; 2 Department of Cardiothoracic Surgery, University Hospital NHS Foundation Trust, Birmingham, United Kingdom; 3 Institute of Medical Sciences, University of Aberdeen, Aberdeen, United Kingdom; Fundação Oswaldo Cruz, Brazil

## Abstract

In the hypertrophied human heart, fatty acid metabolism is decreased and glucose utilisation is increased. We hypothesized that the sarcolemmal and mitochondrial proteins involved in these key metabolic pathways would mirror these changes, providing a mechanism to account for the modified metabolic flux measured in the human heart. Echocardiography was performed to assess *in vivo* hypertrophy and aortic valve impairment in patients with aortic stenosis (n = 18). Cardiac biopsies were obtained during valve replacement surgery, and used for western blotting to measure metabolic protein levels. Protein levels of the predominant fatty acid transporter, fatty acid translocase (FAT/CD36) correlated negatively with levels of the glucose transporters, GLUT1 and GLUT4. The decrease in FAT/CD36 was accompanied by decreases in the fatty acid binding proteins, FABPpm and H-FABP, the β-oxidation protein medium chain acyl-coenzyme A dehydrogenase, the Krebs cycle protein α-ketoglutarate dehydrogenase and the oxidative phosphorylation protein ATP synthase. FAT/CD36 and complex I of the electron transport chain were downregulated, whereas the glucose transporter GLUT4 was upregulated with increasing left ventricular mass index, a measure of cardiac hypertrophy. In conclusion, coordinated downregulation of sequential steps involved in fatty acid and oxidative metabolism occur in the human heart, accompanied by upregulation of the glucose transporters. The profile of the substrate transporters and metabolic proteins mirror the metabolic shift from fatty acid to glucose utilisation that occurs *in vivo* in the human heart.

## Introduction

Under normal physiological conditions, the healthy adult heart derives 60–70% of its energy from the β-oxidation of long chain fatty acids, with the remainder predominantly from carbohydrate sources, such as glucose [1], [2]. Fatty acids are a more energy dense fuel, but require more oxygen for a given amount of ATP formed, when compared with glucose (reviewed in [3]). Therefore, increasing glucose metabolism at the expense of fatty acid metabolism may be beneficial when oxygen is limited. In patients with cardiac hypertrophy, fatty acid utilisation is decreased and glucose utilisation is increased [4], [5]. This metabolic shift is proportional to the extent of cardiac hypertrophy, as fatty acid uptake and oxidation inversely correlate with left ventricular mass and end-diastolic diameter [4], [6], [7].

The underlying mechanisms by which fatty acid utilisation is decreased in cardiac hypertrophy are not fully understood. Biopsies taken from patients with heart failure have reduced mRNA expression of the mitochondrial genes medium chain acyl-coenzyme A dehydrogenase (MCAD), carnitine palmitoyl transferase I (CPT1) and citrate synthase [8], [9], [10], [11]. However, a greater understanding of how metabolic proteins in the various pathways change in relation to each other will give a greater insight into the mechanisms underpinning regulation of *in vivo* metabolic flux in the human heart.

Sarcolemmal fatty acid transporters are the primary regulated step in cardiac fatty acid metabolism. A number of proteins are involved in cardiac fatty acid uptake, including fatty acid translocase (FAT/CD36), plasma membrane and heart-type cytosolic fatty acid binding proteins (FABPpm and H-FABP) [12], [13], [14]. These fatty acid transporters are located at different positions in relation to the sarcolemma; FABPpm associated with the sarcolemmal extracellular surface, FAT/CD36 within the transmembrane region, and H-FABP within the cytosol [13], [14], [15], [16], [17]. The hypothesised mechanism of action of these fatty acid transporters is a co-operative channelling of the fatty acid into the cell [18]. In the heart, FAT/CD36 is hypothesised to be the key regulated step in fatty acid uptake, determining the overall rate of entry into the cardiomyocyte [19], [20]. The role of FAT/CD36 in fatty acid uptake is analogous to that of the glucose transporter GLUT4 in cardiac glucose uptake, both are the primary steps in regulating the metabolism of their respective substrates [21], [22]. However, the relationship between these two opposing substrate transporters and their relationship to disease severity in the human heart has not been investigated.

We have previously demonstrated that FAT/CD36 is downregulated in the failing rat heart, in proportion to fatty acid metabolic rates and contractile function [23]. Here we measured protein levels of the fatty acid and glucose transporters in the hypertrophied human heart, and their relationship to key downstream proteins involved in oxidative metabolism. We hypothesised that FAT/CD36 protein levels would be inversely related to glucose transporter levels, mirroring the metabolic switch from fatty acid to glucose metabolism, and that this would be associated with a downregulation of oxidative metabolic proteins and proportional to disease severity in patients with aortic stenosis.

## Methods

### Patient selection

Patients undergoing elective valve replacement for aortic stenosis, with or without coronary artery revascularisation, were recruited over a period of 6 months (n = 18). The investigation conforms to the principles outlined in the Declaration of Helsinki and recruitment commenced after approval from the South Birmingham Ethics Committee and with full informed written consent from the patients. Patients with complicating diseases, such as diabetes mellitus, were excluded, with the exception of those with hypertension, angina pectoris and hyperlipidaemia. All patients had pure aortic stenosis with no regurgitation.

Pre-operative 2D trans-thoracic echocardiography was performed on all patients. M-mode measurements of septal and posterior wall thickness and left ventricular diastolic diameter, at the level of the chordae tendinae, were taken using a leading edge to leading edge technique. These measurements were used to calculate left ventricular mass using the American Society of Echocardiography cube formula and indexed to body surface area, to give left ventricular mass index (LVMI), an indicator of the degree of hypertrophy. Left ventricular ejection fraction was calculated using the Simpson's biplanar method. Patients were given their usual medications prior to surgery, which included aspirin, statins, β-blockers, angiotensin-converting enzyme inhibitors and calcium channel blockers.

### Sample collection and western blotting

Cardiac biopsy samples were taken from the ventricular apex and atria (n = 18), and samples were immediately frozen in liquid nitrogen. Cell lysates were prepared from the tissue and, for each sample, equal concentrations of total protein were separated on 12.5% SDS-polyacrylamide gels [23]. Gels were incubated in transfer buffer with Immobilon-P membranes (Millipore, UK) and extra thick chromatography paper (BioRad, UK) for 30 min, and transferred using semi-dry transfer apparatus (Biorad, UK) at 0.07 A per gel for 1 h. Membranes were incubated with primary antibodies overnight, diluted in Tris-buffered saline containing 5% (wt/vol) milk powder. FAT/CD36 was detected with the monoclonal antibody MO25, a gift from Dr Narendra Tandon (Otsuka Maryland Medicinal Laboratories, MD) and FABPpm was detected using a primary antibody donated by Dr Jorge Calles-Escandon (Wake Forest University School of Medicine, NC). Primary antibodies raised against H-FABP (also called cFABP) and GLUT1 were purchased from Abcam (Cambridge, UK). Antibodies raised to medium chain acyl-coenzyme A dehydrogenase (MCAD) and α-ketoglutarate dehydrogenase were purchased from Santa Cruz (USA). Prof. Geoff Holman (University of Bath, UK) kindly donated the primary antibody to GLUT4. Antibodies raised to complex I and ATP synthase of the electron transport chain (ETC) were purchased from MitoSciences (USA). Appropriate horseradish peroxidase-conjugated secondary antibodies (Santa Cruz, USA) were diluted 1∶3500 in Tris-buffered saline containing 5% (wt/vol) milk and incubated with the membranes for 2 hours. Bands were quantified using UN-SCAN-IT gel software (Silk Scientific, USA), and all samples were run in duplicate on separate gels to confirm results. Even protein loading between samples and successful transfer were confirmed using Ponceau staining (Sigma, UK). Protein levels were related to internal standards, run on all gels, to ensure homogeneity between samples and gels.

### Fasting plasma metabolites

Blood was collected prior to induction of anaesthesia or administration of heparin, plasma was separated by centrifugation and stored at −80°C. Concentrations of plasma glucose, free fatty acids, triacylglycerol and cholesterol were measured using an automated spectrophotometric analyser (Monarch Laboratories, USA).

### Statistics

Correlations between data sets were calculated using the Pearson correlation coefficient (PASW statistics version 18, USA). All physical, clinical and molecular parameters were correlated with each other, and the significant correlations described in the results. Statistical significance was taken at p<0.05.

## Results

### Clinical characteristics and plasma metabolites

In total, 18 patients were studied, 12 male and 6 female, aged between 48 and 77 years and with a body mass index ranging from 20 to 34 (Table 1). According to the New York Heart Association (NYHA) classification, 10 patients were class I, 5 were class II, 2 were class III and 1 was class IV, and two patients had prior myocardial infarction (one class I and one class IV). Left ventricular mass index, a measurement of the extent of cardiac hypertrophy and disease severity, ranged from 80 to 190 g/m^2^ in the patient group. Left ventricular ejection fraction ranged from 28 to 58%, and there was a strong negative correlation between LVMI and cardiac ejection fraction (r = −0.69, p<0.001). Fasting plasma metabolites are presented in Table 1.

**Table 1:** Patient Characteristics.

**Physical Characteristics**	
Male gender	12/18
Age (years)	69±2
Body weight (kg)	78±4
Body mass index	27±1
NYHA classification I, II, III and IV	55%, 28%, 11% and 6%
Left ventricular mass index (LVMI) (g/m^2^)	126±8
Ejection fraction (%)	50±2
**Fasting plasma metabolites (mmol/l)**	
Free fatty acids	0.73±0.09
Triacylglycerol	1.64±0.23
Cholesterol	3.53±0.37
Glucose	6.32±0.26

### Relationship between fatty acid transporters and mitochondrial metabolic proteins in the human heart

**Figure 1:**
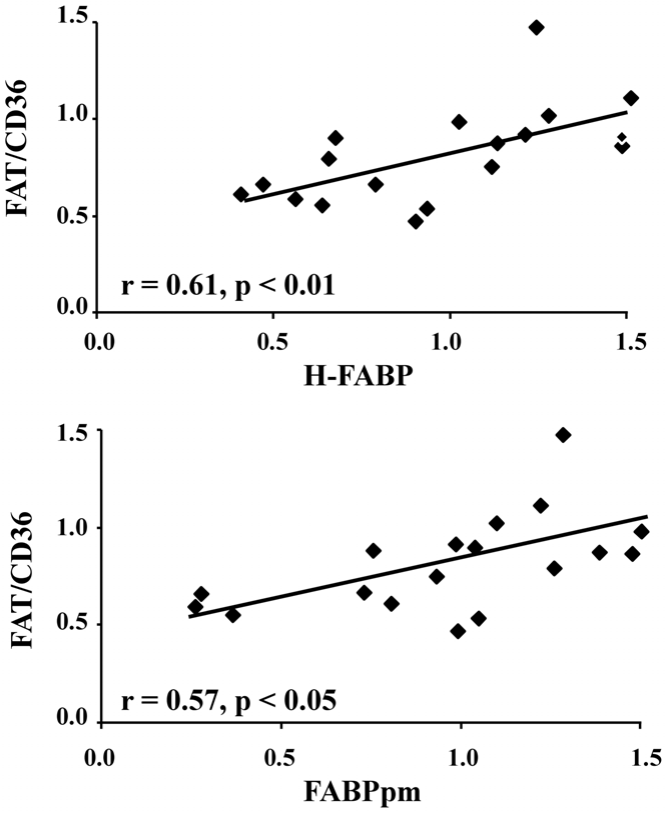
Positive correlations between fatty acid transporters FAT/CD36, H-FABP and FABPpm in the human heart. Protein levels are expressed in arbitrary units, relative to the internal standards.

Relationships were identified between the different fatty acid transporters present in the cardiac biopsies. Positive correlations were found between FAT/CD36 and both H-FABP and FABPpm protein levels (Figure 1) in the atria, demonstrating that changes in the family of fatty acid transporters were co-ordinated.

**Figure 2:**
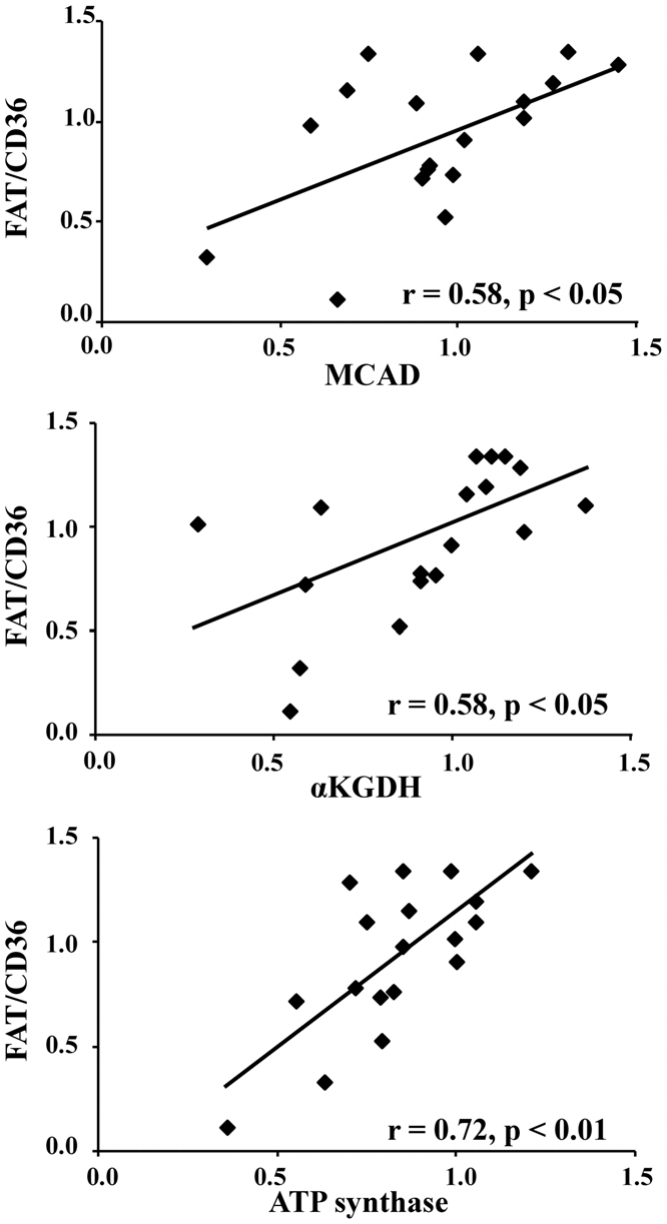
Positive correlations between FAT/CD36 and mitochondrial metabolic proteins including the β-oxidation protein MCAD, the Krebs cycle protein α-ketoglutarate dehydrogenase and ATP synthase in the human heart.

FAT/CD36, the predominant fatty acid transporter in the heart, showed positive relationships with other key downstream proteins involved in mitochondrial oxidative metabolism. Positive correlations were identified between FAT/CD36 and protein levels of MCAD, α-ketoglutarate dehydrogenase and ATP synthase (Figure 2) in the ventricular biopsies. MCAD is a PPARα-regulated protein involved in β-oxidation, α-ketoglutarate dehydrogenase is involved in the Krebs cycle and ATP synthase phosphorylates ADP to ATP. The other fatty acid transporters did not show a significant relationship with the mitochondrial metabolic proteins.

### Relationship between FAT/CD36 and glucose transporters in the human heart

**Figure 3:**
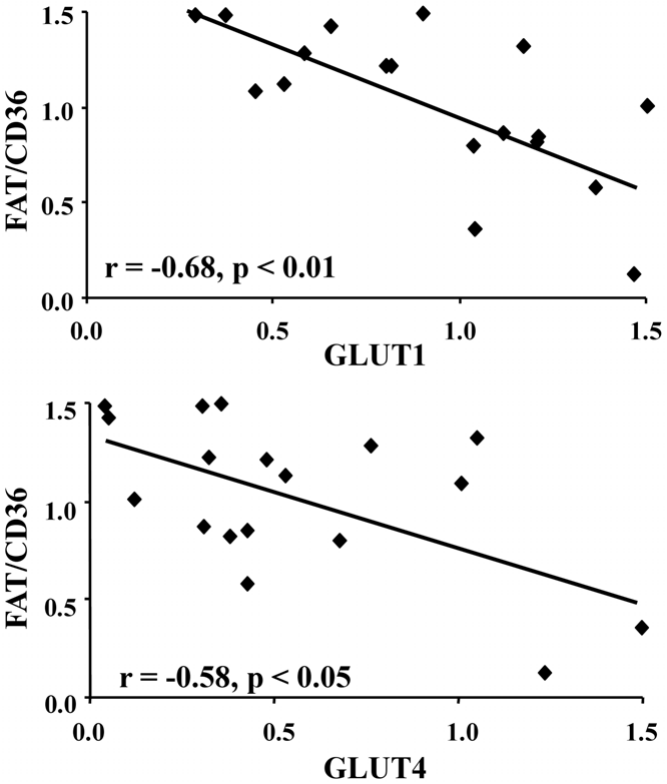
Negative correlation between FAT/CD36 and the glucose transporters GLUT1 and GLUT4 in the human heart.

The heart can switch its substrate preference between fatty acid and glucose metabolism, to provide ATP to power contraction [24]. Negative relationships were identified between FAT/CD36 and the two glucose transporter isoforms expressed in the ventricle (Figure 3). FAT/CD36 correlated negatively with both GLUT1 and GLUT4, thus, in those hearts with the lowest FAT/CD36 protein expression, there was a concomitant upregulation of both glucose transporters. Negative correlations were also found between GLUT4 and both MCAD protein levels (r = −0.62, p<0.01) and FABPpm protein levels (r = −0.57, p<0.05). This data is indicative of the switch between fatty acid and glucose metabolism in the human heart.

### Relationship between metabolic protein and disease severity in the human heart

**Figure 4:**
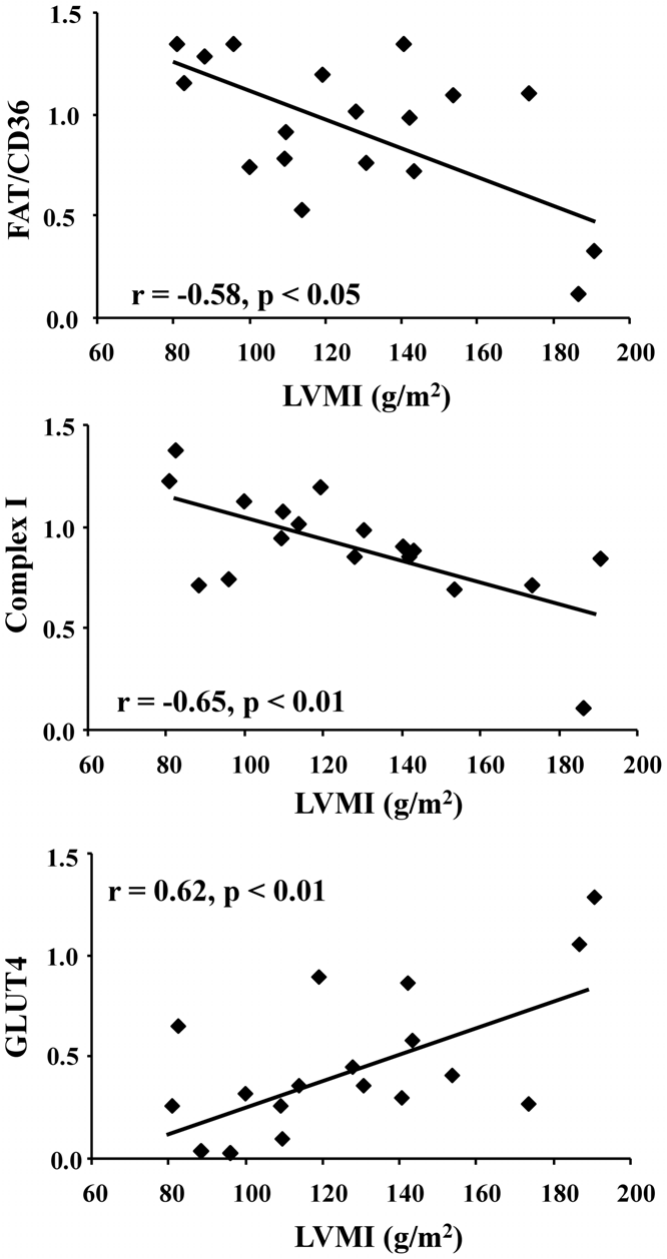
Relationship between metabolic proteins and disease severity in the human heart. Negative correlations between left ventricular mass index (LVMI) and protein levels of FAT/CD36 and complex I of the electron transport chain. Positive correlation between LVMI and GLUT4 protein levels.

In heart disease, the decrease in fatty acid utilisation is proportional to the extent of cardiac hypertrophy [4], [7]. FAT/CD36 correlated negatively with LVMI, thus, patients with the greatest cardiac hypertrophy had the lowest FAT/CD36 protein levels (Figure 4). Similarly, protein levels of electron transport chain complex I correlated negatively with LVMI. This was in contrast to GLUT4, which demonstrated a positive correlation with the extent of hypertrophy. We found no significant correlations between plasma metabolites, extent of coronary artery disease and the metabolic proteins. Thus, with increasing cardiac hypertrophy there was a shift from FAT/CD36 and electron transport chain complex I protein expression, towards GLUT4 protein expression in the ventricle of patients with aortic stenosis.

## Discussion

In this study on the human heart, we have shown that the predominant sarcolemmal fatty acid transporter, FAT/CD36, changed in unison with β-oxidation, Krebs cycle and oxidative phosphorylation proteins, but inversely to the sarcolemmal glucose transporters. The changes in substrate uptake proteins were proportional to disease severity, as an increase in cardiac hypertrophy was accompanied by decreased FAT/CD36 and increased GLUT4. This shift from FAT/CD36 to GLUT4 mirrors the metabolic shift from fatty acid to glucose metabolism that occurs with increasing hypertrophy [4], [7].

The fatty acid transporters have received increasing interest in recent years because of their involvement in the metabolic dysfunction occurring in heart disease, diabetes and lipotoxicity [23], [25], [26]. The positive relationship between FAT/CD36, FABPpm and H-FABP in the present study demonstrates that changes in these three transporters were co-ordinated. Their cellular location indicates they may be involved in sequential steps in the fatty acid uptake pathway, with FABPpm on the extracellular leaflet binding the fatty acid, FAT/CD36 spanning the membrane internalising the fatty acid, and H-FABP within the cytosol transporting the fatty acid away [12], [13], [14]. Thus, a co-ordinated decrease in all three transporters would help regulate the overall process of fatty acid uptake. FAT/CD36 deficiency has been identified in some patients with hereditary hypertrophic cardiomyopathy, associated with a decreased myocardial uptake of fatty acids [27], suggesting that fatty acid transport and metabolism may be intricately linked to cardiac pathology.

*In vivo* measurements of fatty acid metabolism in patients with cardiac hypertrophy have shown decreases in both myocardial fatty acid uptake and oxidation [4], [7]. Studies have reported decreased expression of genes encoding mitochondrial fatty acid oxidation genes, including MCAD and CPT1 [8], [9], [28]. The relative decreases in FAT/CD36, MCAD, α-ketogluturate dehydrogenase and ATP synthase proteins in the present study demonstrates that co-ordinated changes are occurring in sequential pathways involved in fatty acid metabolism, including fatty acid uptake, β-oxidation, the Krebs cycle and oxidative phosphorylation. If, for example, downregulation of fatty acid oxidation occurred independent of a decrease in fatty acid uptake, cytosolic lipid accumulation and lipotoxicity may occur. Thus, synchronised regulation of sequential pathways would ensure deleterious intermediates did not accumulate within the cell. The changes in α-ketoglutarate dehydrogenase and ATP synthase indicate that oxidation of all mitochondrial substrates may be downregulated, not just fatty acids. This is in agreement with the decreased glutamate state 3 respiration rates measured in biopsies from failing human hearts [29], [30]. That the other fatty acid transporters did not display a significant relationship with downstream mitochondrial metabolic proteins was unexpected. However, this could be due to the hypothesised predominant role of FAT/CD36 in regulating the overall fatty acid uptake pathway [19], [20] or due to insufficient statistical power.

The heart has the metabolic flexibility to switch between substrates, both acutely and chronically, according to the physiological or pathological conditions. We have identified a negative correlation between FAT/CD36 and the two glucose transporters, indicating that under conditions when FAT/CD36 is downregulated the capacity for glucose uptake by GLUT1 and GLUT4 is increased. This balance between FAT/CD36 and GLUT4 has been noted in animal models, as in diabetes or following a lipid infusion the expression of FAT/CD36 increases and GLUT4 decreases [31], [32]. In patients with dilated cardiomyopathy, fatty acid uptake is decreased and glucose uptake is increased, indicating that sarcolemmal substrate uptake may be changed in these hearts [4]. Regulating the expression of the sarcolemmal substrate transporters would chronically alter the rate of substrate uptake into the cell and have knock-on effects on downstream substrate metabolism.

In patients with dilated cardiomyopathy, fatty acid uptake rates correlate negatively with left ventricular end-diastolic diameter [4]. The decreasing protein levels of FAT/CD36 with increasing cardiac hypertrophy in the present study provides a mechanism to explain the findings in the former study. Our work on the chronically infarcted rat heart supports these findings, as decreased fatty acid transporter levels were found in the hypertrophied rat heart and were related to decreased fatty acid utilisation and decreased *in vivo* cardiac function [23]. In addition to changes in fatty acid uptake, changes in fatty acid oxidation have also been reported in human heart disease, and correlate with left ventricular mass [7]. Studies on the mitochondrial electron transport chain in the failing human heart by Scheubel *et al* identified selective downregulation of complex I activity, while the other complexes remained unchanged [33]. Similarly, the present study showed that complex I protein levels were selectively decreased in relation to increased cardiac hypertrophy. Taken together, the decreased FAT/CD36 and increased GLUT4 with cardiac hypertrophy resembles the decreased fatty acid metabolism and increased glucose metabolism measured *in vivo* [4], [5]. The substrate transporters may serve to regulate flux into their respective pathways, and aid switching the heart towards a more oxygen efficient fuel under conditions in which oxygen availability may be restricted. If this is the case, these transporters could be attractive therapeutic targets.

A limitation of the present study is that we were unable to include a control group due to lack of availability of biopsy tissue from healthy individuals. However, our data clearly show a relationship between disease progression and metabolic protein levels. In addition, we were unable to distinguish between sarcolemmal and microsomal FAT/CD36, due to small biopsy sizes. However, the total pool of FAT/CD36 has previously been shown to correlate positively with cardiac function and metabolism in the failing rat heart [23].

In conclusion, in patients with aortic stenosis, FAT/CD36 was downregulated whereas GLUT4 was upregulated with increasing cardiac hypertrophy. Decreases in FAT/CD36 were accompanied by decreases in other fatty acid transporters, β-oxidation, Krebs cycle and oxidative phosphorylation proteins, and, in contrast, by increases in both GLUT1 and 4 protein levels. The profile of substrate transporters in the hypertrophied human heart was in agreement with the changes in substrate metabolism measured *in vivo*, suggesting that the sarcolemmal substrate transporters may be involved in the metabolic shift from fatty acid to glucose utilisation in cardiac hypertrophy.
